# Epilepsy services in Saudi Arabia: Quantitative assessment and identification of challenges

**DOI:** 10.17712/nsj.2016.4.20160053

**Published:** 2016-10

**Authors:** Saud M. Alfayez, Bandar N. Aljafen

**Affiliations:** *From the College of Medicine, King Saud University, Riyadh, Kingdom of Saudi Arabia*

## Abstract

**Objective:**

To assess the epilepsy services and identify the challenges in hospitals without epilepsy monitoring units (EMUs). In addition, comparisons between governmental and private sectors, as well as between regions, are to be performed.

**Methods:**

A cross-sectional study conducted using an online questionnaire distributed to the secondary and tertiary hospitals without EMUs throughout the Kingdom of Saudi Arabia (KSA). The study was conducted from September 2013 to September 2015 and regular updates from all respondents were constantly made. Items in the questionnaire included the region of the institution, the number of pediatric and adult neurologists and neurosurgeons along with their subspecialties, the number of beds in the Neurology Department, whether they provide educational services and have epilepsy clinics and if they refer patients to an EMU or intend to establish one in the future.

**Results:**

Forty-three institutions throughout the Kingdom responded, representing a response rate of 54%. The majority of hospitals (58.1%) had no adult epileptologists. A complete lack of pediatric epileptologists was observed in 72.1% of hospitals. Around 39.5% were utilizing beds from internal medicine. Hospitals with an epilepsy clinic represented 34.9% across all regions and sectors. Hospitals with no intention of establishing an EMU represented 53.5%. Hospitals that did not refer their epileptic patients to an EMU represented 30.2%.

**Conclusions:**

Epilepsy services in KSA hospitals without EMUs are underdeveloped.

Epilepsy, which has affected over 50 million people throughout the world, is a chronic non-communicable neurological disease that is characterized by different types of recurrent seizures.^1^ Approximately 7 in every 1000 Saudis suffer from epilepsy. Such common and chronic neurological disorder is associated with a significant social and economic burden to the patients, their families and the healthcare system.^2^,^3^ Additional problems include the challenges related to epilepsy services that epileptics and care providers face. These challenges include the lack of epileptologists, technicians, and funds, among many other obstacles. In this regard, studies in different areas of the world, apart from the Kingdom of Saudi Arabia (KSA), have been conducted to assess epilepsy services and identify these persistent challenges.^4^-^8^ The purpose of this study is to assess the epilepsy services and identify the challenges in KSA. In addition, comparisons between governmental and private sectors, as well as between regions, are to be performed.

## Methods

An institutional review board approval from the College of Medicine at King Saud University, Riyadh, KSA was attained before conducting this cross-sectional study, which targets secondary and tertiary hospitals without epilepsy monitoring units (EMUs) throughout KSA. An online English survey was designed using Google Forms. Items in the questionnaire included the region of the institution, the availability of an EMU, the number of pediatric and adult neurologists and neurosurgeons along with their subspecialties, the number of beds in the neurology department, whether they provide educational services and have epilepsy clinics and if they refer patients to an EMU or intend to establish one in the future. The list of hospitals was obtained from the Saudi Ministry of Health, and neurologists in 80 random hospitals from central, northern, southern, western and eastern regions of the country were contacted through telephone and emails. The objectives were explained to all participants. The study was conducted from September 2013 to September 2015 and regular updates from all respondents were constantly made. After collecting the data, all hospitals with EMUs were excluded from the study. The data was downloaded from Google Sheets as an Excel file. Statistical Package for the Social Sciences Version 21.0 (IBM Corp., Armonk, NY, USA) was used for statistical analysis. Frequencies and percentages were obtained for all data, and the files were split by region and sector (governmental or private) for comparison purposes. The mean, range and standard deviation (SD) were included in the descriptive analysis.

## Results

Forty-three institutions responded, representing a response rate of 54%. The hospitals included in the study were from the western (32.6%), central (27.9%), eastern (16.3%), southern (16.3%) and northern (7%) regions. The responses were from institutions belonging to the governmental (74.4%) and private (25.6%) sectors. The number of adult epileptologists in all hospitals within all sectors and regions ranged from zero to 3 with an average of 0.63±0.9 per hospital. The majority of hospitals (58.1%) had no adult epileptologists including all hospitals in the northern region, while 27.9% had one adult epileptologist. Hospitals with 2 or 3 adult epileptologists were represented by 7% each. **Table 1** shows the distribution of adult epileptologists among the regions. Pediatric epileptologists ranged from zero to 3 with a mean of 0.42±0.76. A complete lack of pediatric epileptologists was observed in 72.1% of hospitals and throughout the northern and southern regions, while 16.3% reported having one pediatric epileptologist. Hospitals with 2 pediatric epileptologists represented 9.3%, and hospitals with 3 pediatric epileptologists represented 2.3%, of the total number of hospitals. **Table 2** demonstrates the distribution of pediatric epileptologists throughout the regions. In terms of adult neurologists, the range was from zero to 8 with an average of 2.47±2 per hospital available in all regions. Most respondents (23.3%) stated that they had 2 adult neurologists employed in their institutions. Hospitals with one neurologist represented 20.9% of the total number of hospitals which is an identical percentage to the hospitals with 3 neurologists. Some hospitals (14%) did not have an adult neurologist, while others reported having 5, 6 and 8 adult neurologists (4.7% each). Pediatric neurologists were present in all regions and ranged from zero to 4 (0.93±1.12). A shortage of pediatric neurologists was stated by 48.8% of the hospitals, which had none. One pediatric neurologist was employed in 23.3%, 2 in 16.3% and 3 in 9.3% of the hospitals. The range of adult neurosurgeons, throughout all regions, was between zero and 8 (2.37±2.09). The majority of hospitals had either 2 or 3 adult neurosurgeons (20.9% each), while the same percentage of hospitals (20.9%) lacked adult neurosurgeons entirely. The mean of pediatric neurosurgeons, across all regions, was 0.33±0.61, ranging from zero to 2. Hospitals without pediatric neurosurgeons were represented by 74.4% and hospitals that had either one were 18.6% or 2 were 7%. Adult epilepsy neurosurgeons were available in the central and western regions and their numbers ranged from zero to 2 (0.12±0.39). Hospitals without adult epilepsy neurosurgeon surgeons were represented by 90.7% and hospitals with one were 7% and hospitals with 2 were 2.3% of hospitals. Pediatric epilepsy neurosurgeons had an average of 0.07±0.26, hospitals, while 93% of the hospitals did not have a pediatric epilepsy neurosurgeon. The remaining 7% had one, and they were all in the central and western regions.

**Table 1:** Reveals the distribution of adult epileptologists among the hospitals in each region of the Kingdom of Saudi Arabia.

Regions	Hospitals with zero to 3 adult epileptologists			
	No adult epileptologist	One adult epileptologist	2 adult epileptologists	3 adult epileptologists
	n (%)			
Central	4 (33.3)	6 (50.0)	1 (8.3)	1 (8.3)
Eastern	3 (42.9)	3 (42.9)	0 (0.0)	1 (14.3)
Northern	3 (100)	0 (0.0)	0 (0.0)	0 (0.0)
Southern	6 (85.7)	1 (14.3)	0 (0.0)	0 (0.0)
Western	9 (64.3)	2 (14.3)	2 (14.3)	1 (7.1)

**Table 2:** Demonstrates the distribution of pediatric epileptologists among the hospitals without an EMU in each region of the Kingdom of Saudi Arabia.

Regions	Hospitals with zero to 3 pediatric epileptologists			
	No pediatric epileptologist	One pediatric epileptologist	2 pediatric epileptologists	3 pediatric epileptologists
	n (%)			
Central	7 (58.3)	3 (25.0)	2 (16.7)	0 (0.0)
Eastern	5 (71.4)	1 (14.3)	1 (14.3)	0 (0.0)
Northern	3 (100)	0 (0.0)	0 (0.0)	0 (0.0)
Southern	7 (100)	0 (0.0)	0 (0.0)	0 (0.0)
Western	9 (64.3)	3 (21.4)	1 (7.1)	1 (7.1)

The number of beds in the neurology departments ranged from 1 to 30; however, 39.5% did not have specific beds as they were utilizing those from internal medicine. Hospitals with an epilepsy clinic represented 34.9% across all regions and sectors. The provision of educational services was reported by 69.8% from both sectors and in all regions.

**Figure 1:**
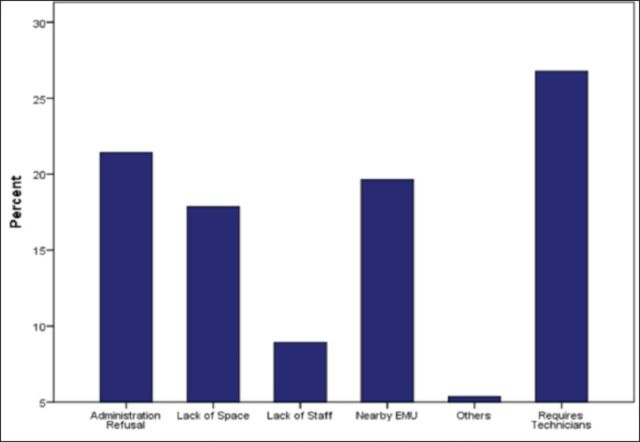
Demonstrates the hospitals’ reasons against establishing an epilepsy monitoring unit in the Kingdom of Saudi Arabia along with the corresponding percentage for each reason.

Hospitals with no intention of establishing an EMU represented 53.5%. The most reported reasons for this were a lack of technicians (60%), administration refusal (48%), a nearby EMU (44%), lack of space (40%), and a lack of epileptologists (20%). Other reasons included the high costs and absence of funds (8%) and that the subject was not discussed with the administration (4%) (**Figure 1**). Regarding the governmental sector, 46.9% did not intend to establish an EMU. The main reason was that it requires technicians (76.6%) followed by the lack of space (58.8%), administration refusal and the presence of nearby EMU (35.3% each). The majority of the private hospitals (72.7%) did not intend to establish an EMU, and their main reasons were administration refusal (75%) and the availability of a nearby EMU (62.5%). The causes of not establishing EMUs in the central region were administration refusal (88.9%), a nearby EMU (66.7%) and the lack of technicians (55.6%). In the eastern region, administration refusal and an available nearby EMU were the main reasons (66.7% each) while the lack of epileptologists in all the northern region hospitals along with the lack of space in half of them were the obstacles behind not establishing an EMU. Furthermore, the reasons in the southern region were due to the lack of technicians (50%), the lack of epileptologists, administration refusal and lack of space (25% each). In the western region, the main preventive factors were the lack of space and technicians (71.4% each).

**Figure 2:**
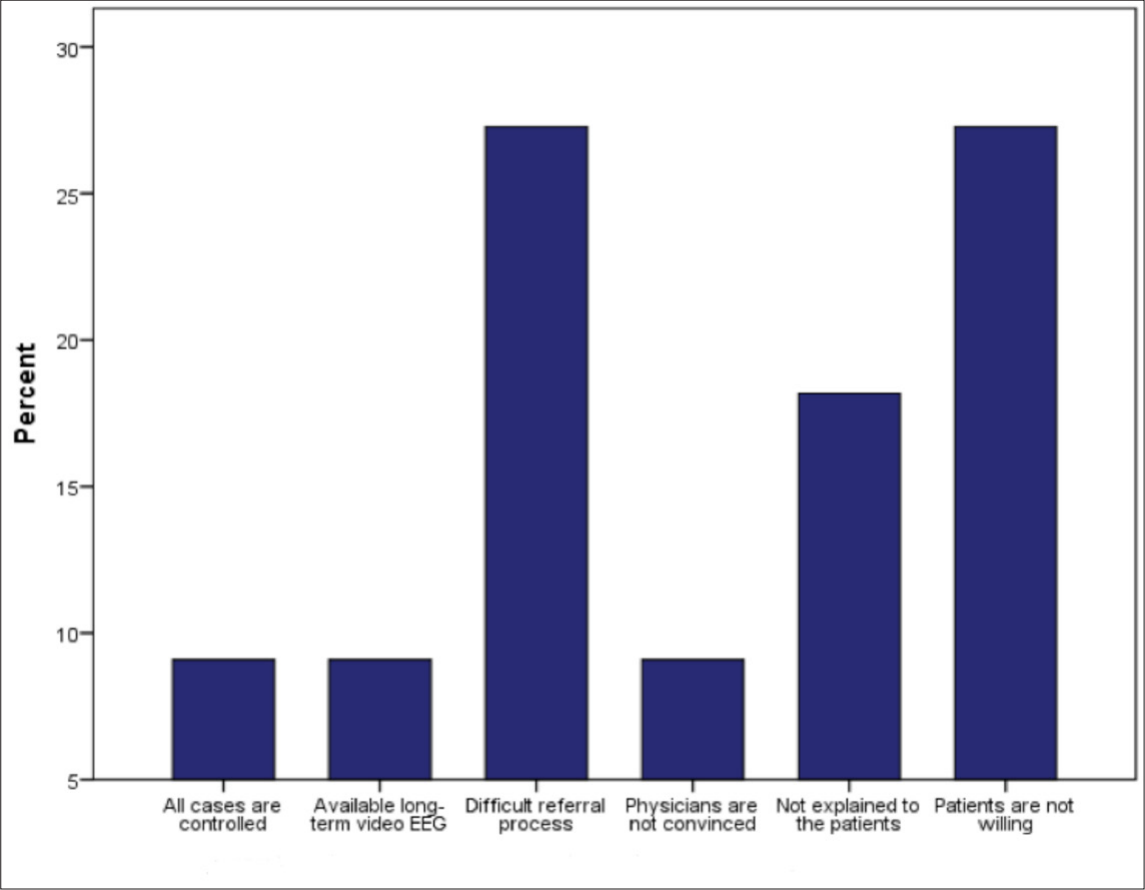
Depicts all the preventive factors for referring patients to an epilepsy monitoring unit in the Kingdom of Saudi Arabia with their corresponding percentages.

Hospitals that did not refer their epileptic patients to an EMU represented 30.2%. The reasons for not referring epilepsy patients to an EMU were the difficult referral process and lack of organization between hospitals and the fact that patients were not willing to be referred (23.3% each). Other factors preventing the referral to an EMU included patients’ lack of understanding of the purpose of the referral (15.4%), the cases were all controlled, the availability of a long term video EEG and that doctors were not convinced to refer to an EMU (7.7% each) (**Figure 2**).

## Discussion

The results indicate that epilepsy services are underdeveloped, especially in the northern and southern regions of the Kingdom of Saudi Arabia in hospitals without EMUs. This is supported by the lack of an epileptologist in the major hospitals in the northern region, while only one adult epileptologist was available in the whole southern region. There was also lack of epilepsy neurosurgeons in both regions. In addition, the majority of hospitals in the northern and southern regions have no intention to establish an EMU, which indicates the persistence of a lack of optimal care for epilepsy patients in the future. On the other hand, most of the eastern institutions were planning to establish an EMU, which shows a positive attitude towards the better provision of epilepsy services in the upcoming years. Although 73% of all epileptologists are practicing in the central and western regions, 75% of central region hospitals, and 64.3% of western region hospitals have no intention of establishing an EMU. A possible reason for this is that, based on findings in this research, most of the EMUs in KSA are located in these 2 regions. The majority of hospitals in both sectors and all regions refer to EMUs, with the exception of the southern region, as 57.1% of the neurologists there did not refer their epilepsy patients. The main preventing factor in that region was that epilepsy patients were not willing to travel to a city outside the southern region where an EMU was available. This reason was reported by 50% of the treating physicians in the south.

Epilepsy incurs a tremendous cost to governments and patients all over the world because continuous follow-ups are needed, and years of compliance to anti-epileptic medications are also required.^9^-^13^ Other burdens associated with epilepsy are frequent hospitalizations, expensive diagnostic tests, and decreased productivity at work for patients or their caregivers. These types of problems can compromise the patients and their families both psychologically and socioeconomically.^14^, ^15^ These difficulties can be minimized by balancing the resources and providing rational epilepsy services in KSA. The EMUs will provide better care for patients and more accurate diagnoses, so hospitals, especially in cities where EMUs are not available, should be financially supported and provided with staff to run an EMU service. Since the most reported obstacle in establishing an EMU was the lack of technicians, incentives should be given to technicians in order to expand EMU presence in KSA. The focus of this study was hospitals without EMUs where most epilepsy patients receive care; however, there has not been a study where the target was hospitals with EMUs in KSA, which should be considered as an idea for future research. One limitation to this study is that the services were not assessed from the patients’ perspectives, which could also be addressed in a future study.

In conclusion, the epilepsy services in the hospitals that lack epilepsy monitoring units are underdeveloped in Saudi Arabia especially in the northern and southern regions of the Kingdom.
